# Development and Validation of Career Choice Competence Scale for High School Students in China

**DOI:** 10.3390/bs16091580

**Published:** 2026-09-07

**Authors:** Yingping Zhang, Weijian Li, Zhe Zhang, Li Chen

**Affiliations:** 1 School of Psychology, Zhejiang Normal University, Jinhua 321004, China; zsdzyp@zjnu.cn (Y.Z.);; 2 Mental Health Center, Junhwa School, Jinhua 321017, China; zhangj@junhwa.cn; 3 Department of Clinical Psychological Science, Maastricht University, 6200MD Maastricht, The Netherlands

**Keywords:** career choice competence, high school students, New Gaokao, scale development, college entrance exam, mixed-methods design

## Abstract

There is currently a lack of validated, culturally embedded measures to assess the career choice competencies that Chinese high school students require for subject and major selection under the New Gaokao system. This study adopted a sequential mixed-methods design to develop and validate such a scale. In Study 1 (*n* = 40), semi-structured interviews were carried out and analyzed, resulting in a preliminary six-dimensional conceptualization of career choice competence. In Study 2 (*n* = 265), item analysis and Exploratory Factor Analysis (EFA) produced a 24-item scale with a seven-factor structure. In Study 3 (*n* = 555 for validation; *n* = 421 for reliability and validity analyses), Covariance Structure Modeling (CSM) confirmed a 24-item final scale with a six-factor structure, comprising self-cognition, learning efficacy, environmental exploration, outcome expectations, self-reflection, and action-taking. Learning efficacy is retained, while Decision-making participation and action-taking are combined into a single dimension: action-taking. As the first study to systematically delineate the psychological meaning and structural dimensions of career choice competence in this population, this study provides a promising assessment tool and offers new perspectives for understanding students’ competence development and advancing career education in China.

## 1. Introduction

Currently, our world is undergoing profound transformation in terms of globalization and technological advancement. This transformation creates an unprecedented level of social volatility, uncertainty, complexity, and ambiguity that is referred to by researchers as the “VUCA Era” (Bennett & Lemoine, 2014). Within this global environment, the dynamic changes taking place in China create a situation in which career choice among young students becomes a personal concern as well as a national priority (Yan, 2025). Therefore, Chinese authorities initiated a significant reform of the National College Entrance Examination in 2014, known as the New Gaokao Reform (X. Wang, 2021). The New Gaokao expands students’ choices of subjects and majors to promote personalized development and overcome the traditional dichotomy between arts and science subjects (Jian, 2020).

In spite of increased student choice within the new reform, the problem of career choice competence still exists. High school students in the context of the New Gaokao in China face anxiety, utilitarian tendencies, and a lack of long-term planning while choosing subjects and majors (Du & Jin, 2016; H. Chen et al., 2021). Although national policymakers highlight the importance of developing students’ ability to make independent career choices (General Office of the State Council, 2019), the career choice process remains inherently complex, involving self and social cognition, comprehension, deliberation, and implementation (Germeijs & Verschueren, 2006). Without systematic support, students are more susceptible to the impact of external factors or are simply unable to make decisions, increasing their career choice anxiety and hindering their career exploration and commitment (Germeijs et al., 2006). Even though some schools have career planning courses, they are poorly integrated into daily education (Gu et al., 2020). Due to family expectations and societal evaluations, students often find it difficult to make intrinsic and independent career choices. This can also limit their subsequent academic pursuits and personal development (Zhang et al., 2018).

### 1.1. The Concept of Career Choice Competence

The conceptual development of career choice competence can be viewed against the background of a broader paradigm shift in understanding career development from a static model to a more dynamic, lifelong process of meaning-making. The last century of research in the field of career development was marked by three shifts in theoretical assumptions about it: from the trait-factor matching model (Parsons, 1909) to developmental models (Super, 1980; Creed & Patton, 2003) and further to constructivist and process-oriented models (Savickas, 2013; Lent & Brown, 2013). However, each theoretical development has come out of changing socio-economic circumstances, and now it becomes necessary to reconsider the very concept of “competence” for making career decisions due to an increasingly uncertain society.

According to Savickas (2013), postmodern conditions of working life imply that “job security is history”. Career development is no longer a linear process that starts in school and ends with obtaining stable employment. Instead, it becomes a lifelong process of constructing oneself and making sense of one’s career through ongoing interaction with the environment (Savickas, 2013; Hirschi et al., 2018). Under these circumstances, career choice competence cannot be understood as an ability to make a “correct” decision on a particular occasion. It rather means a process-oriented, adaptive, and reflexive ability to make career decisions in changing circumstances. Career competencies include “reflective, communicative, and behavioral” dimensions that allow people to navigate the boundary between their personal aspirations and external constraints (Akkermans et al., 2018). Thus, career choice competence is not a static, one-time ability but a capacity that enables individuals to exercise agency over their career trajectories in an uncertain world.

The concept of career choice competence of high school students arises from the practice of career education in high schools within the New Gaokao reform in China. As provinces or cities at the vanguard of the New Gaokao reform, Shanghai and Zhejiang have made pioneering explorations in the field of career education. For instance, Jinyuan High School, which has a history of over one hundred years, in Shanghai, is dedicated to fostering students’ career choice competence in accordance with the principle of “choice education” (Liu & Zhang, 2019). Likewise, Zhejiang Lishui High School believes that career education is not simply about subject or major selection; rather, it should cultivate students’ “choice competence” so as to make them autonomous learners (S. R. Fan et al., 2018).

Although the concept of career choice competence originated from Western theoretical frameworks, it was gradually localized in the context of China’s educational system. The localized concept pays special attention to the integration of personal aspirations, external conditions, and the broader environment, and is characterized by a high degree of integration, a forward-looking perspective, and strong contextual awareness rooted in traditional Chinese culture. This paper defines Career Choice Competence as the comprehensive psychological competence of high school students in processing and generating information regarding themselves and their surrounding environment when facing dynamic career choice tasks. It allows students to participate in and lead the decision-making process, helping them continuously explore their career direction, reach prudent decisions, and adapt flexibly to changes.

### 1.2. Evolution of Structural Dimensions of Career Choice Competence

To clarify the conceptual boundaries of career choice competence (CCC), it is necessary to distinguish it from three related yet distinct constructs in the career literature: career decision-making self-efficacy (CDMSE), career choice readiness (CCR), and career adaptability (CA). Table 1 summarizes their theoretical foundations, core definitions, and dimensional structures.

As shown in Table 1, CDMSE captures perceived confidence in decision-making; CCR focuses on static preparedness prior to decision-making; CA serves as a general life-span resource for adapting to various career changes. However, the students’ career choice process under China’s new Gaokao system exhibits two distinctive features: time constraints and a collective cultural nature. Most students must complete subject selection before the end of their first year of high school, followed by university major selection after the exam, and this choice is often made under collective family decisions and social pressures. Compared with the individualistic career choice systems in Western countries, career choice among Chinese high school students requires more comprehensive consideration of individual-family relationships, personal interests, and societal demands.

### 1.3. Social Cognitive Career Theory (SCCT)

The theoretical basis of this study is Social Cognitive Career Theory (SCCT; Lent et al., 1994). According to SCCT, career development is influenced by the complex interaction between personal characteristics, the environment, and behavioral experiences. Three key cognitive processes—self-efficacy beliefs, outcome expectations, and personal goals—play a crucial role in such an interaction, influencing interest development, choice goals, choice actions, and performance outcomes. Although SCCT has already been extensively used in Western countries and among university students, its use in high school settings among students from non-Western education systems—especially under high-stakes and time-constrained choice conditions—has rarely been explored (Lent et al., 2018; Ding, 2021). The present study draws on SCCT to conceptualize career choice competence as a process-oriented competence that enables students to operationalize these social-cognitive mechanisms in the specific context of China’s New Gaokao reform.

Other process-oriented tools, such as the Study Choice Task Inventory (SCTI), assess student progress in academic career selection across five factors: (1) orientation, (2) self-exploration, (3) broad environmental exploration, (4) in-depth environmental exploration, and (5) decision status (Germeijs & Verschueren, 2006). Driesel-Lange developed a career choice competence model that categorizes relevant abilities into four sequential stages: Tuning in, Exploring, Deciding, and Attaining (Klein et al., 2022). These existing findings have deepened scholarly understanding of career choice competence research.

Despite these advances, existing instruments lack contextual alignment with specific educational contexts, such as that of China under the New Gaokao reforms. Recent scholarship highlights the need to integrate personal agency with the psychosocial environmental factors (Z. Wang et al., 2025), and develop a localized assessment tool that reflects the characteristics of China’s educational environment and the development of high school students’ career choice competence (Ding, 2021). It aims not to evaluate choice outcomes, but to foster students’ awareness and proactive enhancement of their autonomous choice competence throughout the career choice process.

### 1.4. This Study

Given the currently limited research on career choice competencies among Chinese high school students, and particularly the severe lack of assessments that have undergone scientific, indigenous revision and generated local norms (W. Fan & Leong, 2016)—a gap rooted in the absence of culturally adapted theoretical models tailored to local contexts—this study aims to develop and validate a new scale via a mixed-method design that combines qualitative and quantitative approaches. It intends to provide a culturally and contextually appropriate instrument for measuring these competencies in the specific context of China’s New Gaokao reform.

The research involves three major studies. First, qualitative techniques such as in-depth interviews with high school students in China will be utilized in order to understand their process of making career decisions under the New Gaokao reform. Moreover, the psychological structure of career choice competence will be uncovered. Second, a preliminary instrument will be developed and refined through item analysis and Exploratory Factor Analysis (EFA). Third, Covariance Structure Modeling (CSM), which is a type of Structural Equation Modeling (SEM), will be used in order to confirm the factor structure and assess the reliability and validity of the measure. The final outcome will be a standardized and psychometrically sound assessment tool for measuring career choice competence in the context of China’s New Gaokao reform.

## 2. Method

### 2.1. Participants

Sample for study 1: Using the purposive sampling method (X. M. Chen, 2000), 40 high school students (Guest et al., 2006; Wutich et al., 2024) were selected from six schools in four cities of Zhejiang Province. In total, there were 21 boys and 19 girls, between 15 and 18 years old: seven from Grade 10, 16 from Grade 11, 17 from Grade 12, 34 from key schools and six from regular schools, and 21 from municipal-level institutions and 19 from county-level institutions.

Sample for study 2: In the present research, 285 high school students were chosen from two high schools in Zhejiang Province. Excluding 20 invalid questionnaires, 265 valid ones remained, which means the validity rate was 93.0%. In the end, the sample contained 130 boys and 135 girls between 14 and 18 years old (M = 16.15).

Sample for study 3: There were two independent samples involved in this study. Sample A (*n* = 555), chosen from three high schools, had 272 boys and 283 girls between 15 and 18 years old (M = 16.16). Excluding 21 invalid questionnaires from the initial 576 questionnaires, the valid response rate was 96.35%, and this sample was used for Covariance Structure Modeling (CSM). Sample B (*n* = 421) was from two additional high schools and had 185 boys and 236 girls between 15 and 19 years old (M = 16.33). After 34 invalid cases were excluded from the initial 455 participants, the valid response rate was 92.53%, and this sample was used to assess reliability and validity.

### 2.2. Measures

#### 2.2.1. Career Choice Competence Scale (CCCS) for High School Students

In Study 3, the 24-item Career Choice Competence Scale (Appendix A Table A11) was employed to test the reliability and concurrent validity of the scale among high school students. This scale includes six dimensions: self-cognition (3 items), learning efficacy (4 items), environmental exploration (4 items), self-reflection (4 items), outcome expectations (3 items), and action-taking (6 items).

#### 2.2.2. Career Adapt-Abilities Scale (CAAS)

The short form of the Career Adapt-Abilities Scale, which consists of 12 items covering four dimensions (career concern, career control, career curiosity, and career confidence), was used to examine its correlation with the CCCS among high school students. The items are scored on a 5-point scale ranging from 1 to 5, with higher scores indicating better career adaptability. The scale demonstrates good reliability, construct validity, and criterion-related validity (Maggiori et al., 2017). In this study, the internal consistency reliability (Cronbach’s α) of this scale was 0.873.

### 2.3. Procedure and Data Analysis

#### 2.3.1. Study 1: Procedure and Analysis for the Qualitative Exploration

Semi-structured interviews were conducted to explore three core themes: (1) high school students’ current career choice status under the New Gaokao system, including subject selection, major choice, and considerations for future careers; (2) students’ career decision-making processes (e.g., “How do you choose your subjects?” and “How do you determine whether this choice is right for you?”); and (3) students’ self-assessment of their own career choice competence. The complete interview protocol is presented in Appendix A (Table A1). Interviews were conducted either face-to-face or by telephone under the guidance of a structured interview protocol. Each session lasted between 20 min and one hour. With participants’ informed consent, all interviews were documented through written notes and audio recording. All recorded content met quality standards and was transcribed verbatim.

In order to conduct an analysis of the interview data (102,500 Chinese characters), grounded theory (X. M. Chen, 2000) was used in this study. This methodology creates theoretical constructs through an iterative inductive-deductive reasoning process. The analysis was conducted through the following three coding processes: open coding (first-level coding), axial coding (second-level coding), and selective coding (third-level coding), whereby dimensions were progressively extracted, and a theoretical model was developed (X. M. Chen, 2000). The coding process was facilitated with the help of the software program NVivo 12.0, which provides a complete Chinese-language user interface and has become widely used in qualitative research within China (Pan & Tang, 2020). The coding was conducted by two independent coders through negotiated coding (Garrison et al., 2006; Hemmler et al., 2022). Both coders are doctoral candidates in psychology with formal training in qualitative methods. At each coding stage, the coders held targeted discussions to clarify coding definitions and ensure coding reliability. A complete codebook, including an example of the conceptualization process for open coding and the distribution of coding nodes for the psychological connotation of career choice competence, is provided in Appendix A (Table A2 and Table A3).

We employ the classification agreement index, which is defined as the percentage of cases in which raters performed identical content analysis, coding, and categorization for the same interview transcripts relative to the total number of coded entries: CA = 2 × (T_1_ ∩ T_2_)/(T_1_ ∪ T_2_). Here, T_1_ represents the number of codes assigned by Rater 1, and T_2_ represents the number of codes assigned by Rater 2 (X. M. Chen, 2000). After the consensus process, we calculated that the initial inter-coder agreement after negotiation was 87.3%. We assessed saturation using inductive thematic saturation by tracking the emergence of new codes after each interview, and found that no new codes emerged after the 35th interview. The remaining five interviews were accordingly used to confirm the saturation we reached (Hennink & Kaiser, 2022).

The final coding structure was validated using the Delphi expert consultation method (Hasson & Keeney, 2011), and the consistency of expert ratings was evaluated via Q-values (interquartile range, IQR). Q < 1 indicates high consistency, and Q < 1.8 indicates acceptable consistency (J. Wu & Huang, 2012; B. Wang & Hu, 2016). All ten experts assigned Q-values below 1.8 to all secondary indicators, indicating an acceptable level of consensus.

#### 2.3.2. Study 2: Procedure and Analysis for Scale Development

This study adopted a paper-based questionnaire survey design. Participants completed the 50-item initial version of the Career Choice Competence Scale (CCCS), which covers six dimensions. All items were rated on a 5-point Likert scale ranging from 1 (strongly disagree) to 5 (strongly agree), and the scale included four reverse-scored items (Items 33, 37, 41, and 49). All reverse-scored items were recoded prior to item analysis and EFA (i.e., responses were inverted so that higher scores consistently reflected higher perceived competence). We used SPSS 25.0 to conduct item analysis and Exploratory Factor Analysis (EFA) with PAF extraction and Promax rotation, and eliminated items with poor psychometric performance or weak model fit.

Based on the item selection criteria proposed by M. Wu (2010), the questionnaire items selection were required to meet the following conditions: ① Conduct an extreme-group comparison by ranking the total questionnaire scores from high to low, selecting the top 27% and bottom 27% values as the cutoff points for the high-and low-score groups, and using an independent samples *t*-test to examine the discrimination power of the high-versus low-score groups for each item (*p* < 0.05) as well as the decision criterion (t ≥ 3.00); ② Items should show a significant correlation with the total questionnaire score (*p* < 0.05, r > 0.40), and those that do not meet this criterion will be removed accordingly. ③ Homogeneity test: if removing a specific item increases the overall reliability coefficient of the scale compared to the value before removal, this indicates that the attribute or psychological trait measured by this item may be inconsistent with what the other items measure. Consequently, this item should be removed due to its low homogeneity with other items. These thresholds were applied as a substantive criterion, rather than relying solely on a test of statistical significance.

#### 2.3.3. Study 3: Procedure and Analysis for Scale Reliability and Validation

Given that model modification was conducted based on empirical fit indices, this study employs Covariance Structure Modeling (CSM; M. Wu & Zhang, 2013) rather than purely confirmatory factor analysis. This approach acknowledges the exploratory element inherent in model modifications (MacCallum et al., 1992). The researchers administered the 24-item CCCS to Sample A and performed Covariance Structure Modeling (CSM) analysis using Amos 24.0. Given the discrepant results between the qualitative findings in Study 1 and the Exploratory Factor Analysis (EFA) in Study 2, two models were tested: a seven-factor model based on the EFA results was set as the hypothesized model (M0), while a six-factor model was set as the competing model (M1). Sample B completed both the CCCS and the Career Adapt-Abilities Scale to assess internal consistency reliability, the criterion-related validity and cross-validation of the CCCS.

## 3. Results

### 3.1. Study 1: Exploring the Psychological Structure of Career Choice Competence

#### 3.1.1. Open Coding

With the assistance of NVivo 12.0, the researchers conducted in-depth reading and semantic analysis of the interview transcripts (X. M. Chen, 1999), and iteratively identified, compared and integrated repeated meaning units related to “career choice competence”. Through this process, 52 open coding nodes with 584 reference points were extracted (see Appendix A Table A3). The sample achieved theoretical saturation, as no new codes emerged after the 35th interview.

#### 3.1.2. Axial and Selective Coding

Through axial coding, the 52 initial codes were systematically compared and integrated into 20 higher-level concepts based on their semantic relationships. Subsequent selective coding further consolidated these categories into six core dimensions: self-exploration, environmental exploration, outcome expectations, decision-making participation, self-reflection, and action-taking (see Appendix A Table A2 and Table A3).

The six dimensions reflect three progressive levels of career choice competence: (1) foundational exploration abilities (self-exploration and environmental exploration); (2) core decision-making capabilities (outcome expectations and decision-making participation); and (3) advanced self-management abilities (self-reflection and action-taking). Interview data indicated varying competency levels among participants. The majority of students are at the exploratory stage of career choice competence; some developed outcome expectations and decision-making skills when facing selection tasks; and only a few students have developed higher-level career management abilities, specifically self-reflection and action-taking skills.

### 3.2. Study 2: Development of the Career Choice Competence Scale

#### 3.2.1. Item Analysis

Based on participants’ total scores on the career choice competence questionnaire, we divided respondents into a high-competence group (the top 27%) and a low-competence group (the bottom 27%), and conducted an independent samples *t*-test on the item-level scores of the two groups. The results showed that, except for 2 items, all other items exhibited significant differences between the high and low groups, indicating high discrimination. Furthermore, the item-total correlation test revealed that 12 items did not meet the significance standard and were removed (item-total correlation of r < 0.40). After examining the reliability, three of these 12 items had an alpha value below the scale’s overall reliability (α = 0.938). Following item analysis, 12 items did not meet the standard indices and were removed (see Table A4).

#### 3.2.2. Exploratory Factor Analysis

Following the recommendations of Fabrigar et al. (1999), this study conducted Exploratory Factor Analysis (EFA) with Promax rotation as a data-reduction technique to explore the dimensional structure of the construct. EFA is appropriate for the exploratory purpose of this phase, particularly given that subsequent confirmatory validation will be conducted in Study 3. Bartlett’s test of sphericity (χ^2^ = 5588.357, df = 703, *p* < 0.001) and the KMO test (KMO = 0.904) indicated that common underlying factors likely existed among the items, confirming that EFA was appropriate (Dziuban & Shirkey, 1974). In performing the exploratory factor analysis with Promax rotation, the study determined the number of factors based on the criteria of eigenvalues greater than 1 and visual inspection of the scree plot. Following previous research standards, two types of items were eliminated: (1) items whose absolute factor loading was less than 0.4 on all factors; and (2) items with absolute factor loadings on multiple factors where the difference between the loadings was less than 0.15. After each elimination step, EFA was re-run until no items met either elimination criterion. After three rounds of exploratory factor analysis (EFA), 14 items were eliminated: (five items were removed because their absolute factor loadings were lower than 0.4; seven items (Item X4, X16, X20, X24, X25, X26, X27) were eliminated (because the difference between their cross-loadings on multiple factors was less than 0.15; and the remaining two items (Item X14, X15) were removed due to poor fit with extracted factors. Finally, 24 items were retained, distributed across seven factors, and these seven factors cumulatively explained 63.901% of the total variance. The factor loading of each item on its corresponding factor was substantial, ranging from 0.45 to 0.88 (see Table A5 in Appendix A).

Through Exploratory Factor Analysis (EFA), this study developed a 24-item career choice competence assessment scale. This scale covers seven dimensions: self-cognition (3 items), learning efficacy (4 items), environmental exploration (4 items), outcome expectations (3 items), decision-making participation (3 items), self-reflection (4 items), and action-taking (3 items). Different from the results of prior qualitative research, the self-exploration dimension identified in the qualitative study was subdivided into two separate dimensions: self-cognition and learning efficacy.

### 3.3. Study 3: Reliability and Validation of Career Choice Competence Scale

#### 3.3.1. Covariance Structure Modeling (CSM) Analysis

Model fit was evaluated and compared using the following fit indices: the chi-square to degrees of freedom ratio (χ^2^/df), comparative fit index (CFI), Tucker–Lewis index (TLI), root mean square error of approximation (RMSEA), and standardized root mean square residual (SRMR) (Hu & Bentler, 1999). By convention, a χ^2^/df value < 5, CFI and TLI values > 0.90, and an RMSEA value < 0.08 indicate an acceptable model fit. We also assessed the reasonableness of parameter estimates: factor loadings above 0.70 are considered good, while loadings below 0.50 usually require the corresponding item to be removed (Hair et al., 2011).

Fit indices for the initially hypothesized seven-factor model (M0) are presented in Table 2. The model showed marginally acceptable fit according to some criteria (χ^2^/df = 3.437; RMSEA = 0.066) but poor fit according to others (CFI = 0.884, TLI = 0.871), both of which fell below the recommended threshold of 0.90. Furthermore, discriminant validity analysis revealed a very high correlation between the “decision-making participation” and “action-taking” factors (r = 0.760), suggesting the two may represent a single construct. Consequently, M0 was rejected.

A competing six-factor model (M1) was subsequently tested. This model was formed by merging the two highly correlated factors and removing several items with low factor loadings. As shown in Table 2, the fit indices for M1 were excellent: χ^2^/df = 2.677, CFI = 0.950, TLI = 0.941, and RMSEA = 0.055. All indices met or exceeded established thresholds for good model fit. Therefore, the hypothesized seven-factor model (M0) was rejected in favor of the better-fitting six-factor model (M1).

Using the maximum likelihood estimation method in AMOS, we conducted covariance structure modeling. The results supported a six-factor structure, confirming the hypothesized dimensions of the scale. All correlations between the factors were statistically significant, providing initial evidence of adequate construct validity.

To further evaluate the measurement model, we assessed both convergent and discriminant validity. Convergent validity was confirmed: the composite reliability (CR) of all six factors exceeded 0.7, and all standardized factor loadings were greater than 0.5 (Fornell & Larcker, 1981) (see Table A6 in Appendix A). Discriminant validity was supported by the moderate inter-factor correlations. As shown in Table A7 in Appendix A, the square roots of the average variance extracted (AVE) values (the bolded diagonal entries) are all larger than the correlation coefficients between each dimension and all other dimensions (the off-diagonal entries). This indicates that all dimensions of the scale have good discriminant validity.

**Figure 1:**
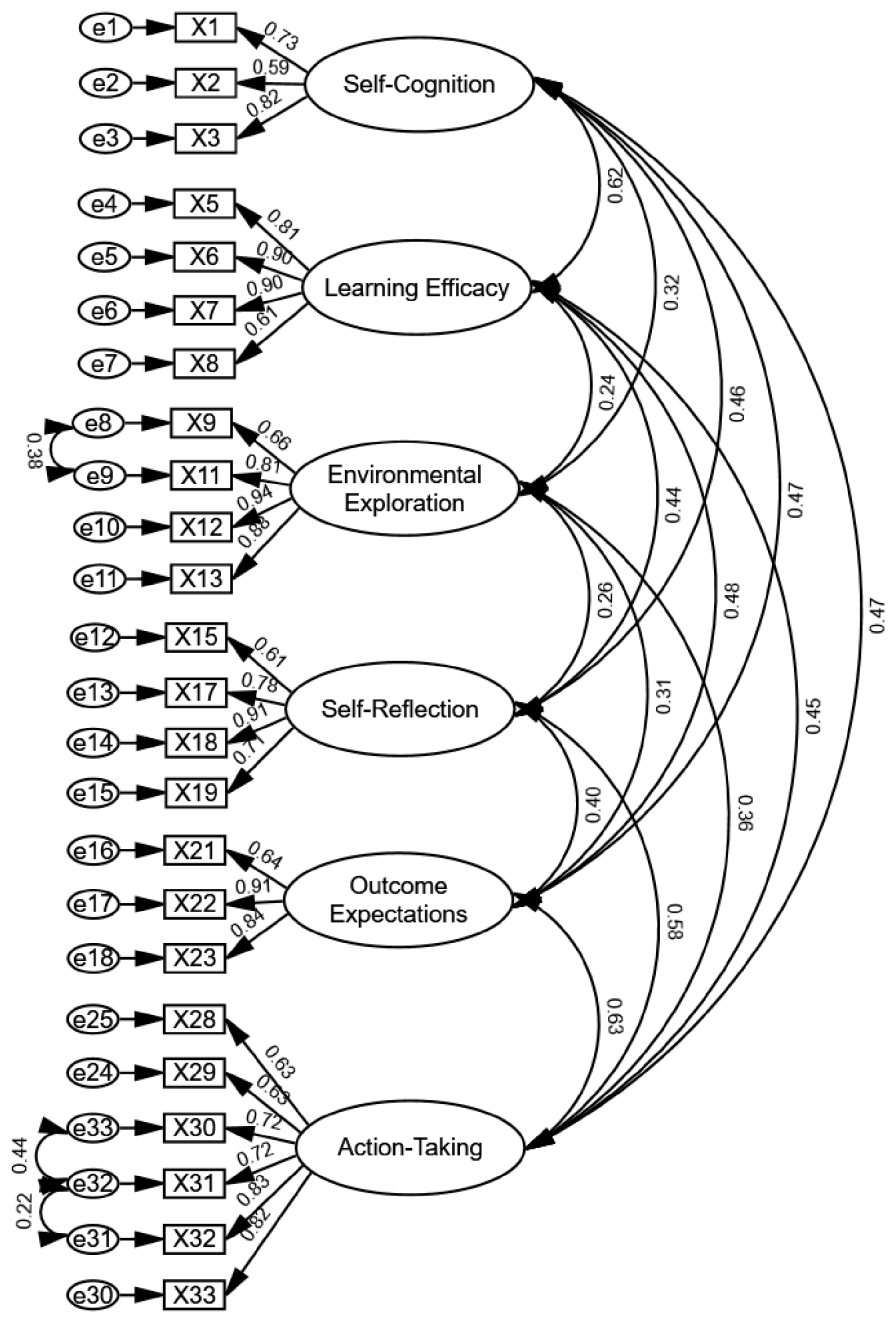
Six-factor model confirmatory factor analysis results.

Taken together, these results confirm that career choice competence is a six-dimensional construct, as presented in the final structural model (see Figure 1).

#### 3.3.2. Reliability Analysis

We examined the reliability of the 24-item CCCS for high school students, using Cronbach’s α for both the full scale and its six sub-dimensions. As shown in Table 3, the overall scale has excellent internal consistency (α = 0.932). The reliability coefficients of the six sub-dimensions range from 0.759 to 0.912, which indicates that all constructs have good internal consistency.

#### 3.3.3. Evidence of Cross-Validation and Validity Based on Relations with CAAS

To assess validity, we conducted a cross-validation of the final six-factor model in the independent Sample B (*n* = 421). The model demonstrated acceptable fit: χ^2^/df = 2.707, CFI = 0.937, TLI = 0.926, RMSEA = 0.064, supporting the stability of the six-factor structure (see Table A9 in Appendix A). Next, the 12-item short-form Career Adapt-Abilities Scale (CAAS; Maggiori et al., 2017) was selected as a theoretically relevant criterion measure. In this study, we evaluated concurrent validity by examining the relationship between the CCCS and the simultaneously administered CAAS. A strong positive correlation would offer preliminary evidence supporting the validity of the CCCS based on its association with a theoretically related variable, though we recognize this is only one component of the broader validation process.

We hypothesized that CCCS scores of high school students would show a significant positive correlation with CAAS scores. The internal consistency coefficients for all sub-dimensions of the two scales fell between 0.642 and 0.912 (as shown in Appendix A, Table A8). As shown in Table A10 in Appendix A, Pearson correlation analysis detected a strong, statistically significant association between the total scores of the two scales (r = 0.736, *p* < 0.01). The correlations between each of the six competence sub-dimensions of CCCS and the total CAAS score ranged from 0.374 to 0.652, and all these correlations were significant at the *p* < 0.01 level. Statistically significant correlations were also found between the corresponding sub-dimensions of the two scales, with correlation coefficients ranging from 0.229 to 0.554 (*p* < 0.01).

These results demonstrate a consistent and statistically significant relationship between CCCS and CAAS, which provides preliminary evidence for the validity of the Career Choice Competence Scale developed for high school students.

## 4. Discussion

The present study employed a sequential mixed-methods design to develop and validate a Career Choice Competence Scale (CCCS) for Chinese high school students within the context of the New Gaokao reform in China. By integrating qualitative exploration (Study 1), scale development with Exploratory Factor Analysis (Study 2) and Structural Equation Modeling (Study 3), this study constructed a psychometrically valid six-dimensional model of career choice competence. In this general discussion, we synthesize the findings across the three studies.

### 4.1. The Measurement Model of Career Choice Competence

The qualitative results of Study 1 produced a preliminary six-dimensional framework covering self-exploration, environmental exploration, outcome expectations, decision-making participation, self-reflection, and action-taking. EFA in Study 2 revealed a seven-dimensional structure, with the dimension “self-exploration” empirically distinguished into “Self-cognition” (knowledge of one’s traits, interests and abilities) and “Learning Efficacy” (students’ perceived interests in specific subjects, perceived ease of learning, and reasoning strengths). The CSM conducted in Study 3 validated a final six-factor structure: self-cognition, learning efficacy, environmental exploration, outcome expectations, self-reflection, and action-taking.

**Figure 2:**
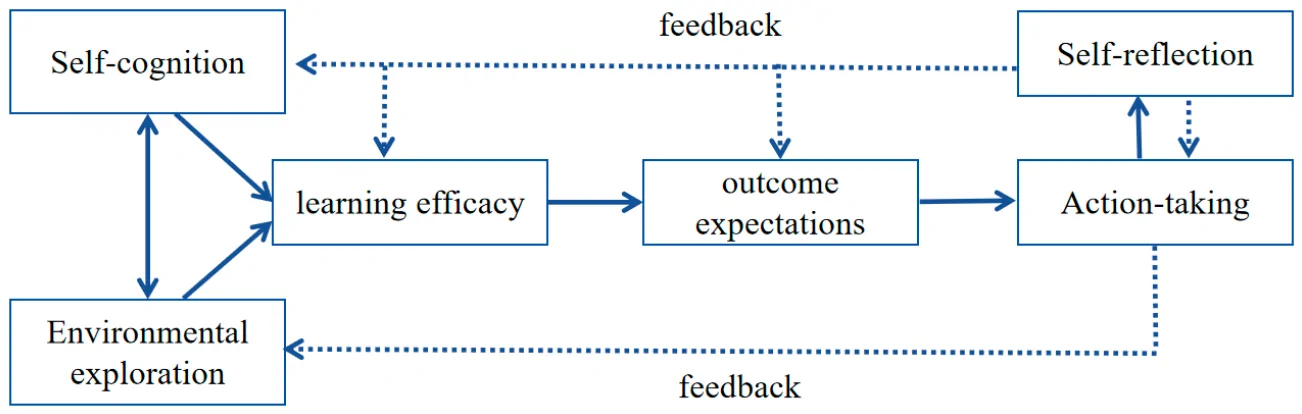
Theoretical process model of career choice competence (solid arrows depict the forward progression from exploration to action, whereas dashed arrows illustrate the reflective feedback mechanism that enables ongoing adaptation throughout the career choice process).

Figure 2 presents the theoretical process model of career choice competence based on the final six-factor structure, and it aligns with and extends the Social Cognitive Career Theory (SCCT; Lent et al., 1994). The directional relationships depicted are theoretical propositions derived from SCCT and the qualitative findings, rather than empirically tested causal paths. Self-cognition and learning efficacy correspond to personal input and self-understood mechanisms; environmental exploration reflects contextual affordances and information-gathering; outcome expectations and action-taking map onto the goal-setting and choice-action components. This pathway aligns with the core sequence of social cognitive theory, in which beliefs and explorations shape goals and the subsequent actions taken to achieve them (Lent et al., 1994). Theoretically, learning efficacy is not identical to the defined academic self-efficacy in SCCT (Lent et al., 1994), which focuses specifically on students’ understanding and evaluation of their ability to polish their learning process (Chao & Liang, 2025). Rather, our items capture a broader perception of the learning process itself. It is conceptually linked to outcome expectations and action-taking within the SCCT framework (Hui & Lent, 2018). However, given the cross-sectional nature of this study, this relationship should be interpreted as a theoretical proposition; future longitudinal research is needed to test the directional relationship among these dimensions.

Moreover, including self-reflection as a core dimension adds a meta-cognitive layer to the career choice process. Compared with the Study Choice Task Inventory (SCTI; Germeijs & Verschueren, 2006), the CCC model focuses on measuring “processes” such as exploration and decision-making rather than merely “outcomes”. The factor structure of career choice competence indicates that conscious reflection on one’s choice processes, abilities, and outcomes is a measurable and integral component of competence (see Figure 1). This is consistent with the constructivist approach to career development, which emphasizes the process of meaning-making and internal dialogue in adaptive career behaviors (Savickas, 2013, 2005).

The combination of “decision-making participation” and “action-taking” into one final dimension can offer some theoretical insights, implying that for adolescents under the New Gaokao system, decision-making and action-taking behaviors are psychologically intertwined. Making a choice, such as selecting an academic track, immediately requires corresponding concrete preparatory actions, differing from contexts where exploration and choice may be temporally separated from subsequent actions.

### 4.2. Distinctions from Related Career Constructs

The CCCS demonstrates clear empirical and conceptual distinctions from related career constructs. The most fundamental difference between CDMSE (career decision-making self-efficacy) and CCCS lies in the distinction between confidence and competence: CDMSE captures an individual’s confidence in performing career decision-making tasks (Taylor & Betz, 1983), whereas the CCCS assesses self-reported career choice competence—that is, students’ perceptions of their own competencies across six dimensions.

The CCCS differs from CCR (Career Choice Readiness) in its focus on process versus preparedness. Rooted in the Cognitive Information Processing (CIP) theory (Sampson et al., 2000), CCR emphasizes cognitive, affective, and social preparedness prior to decision-making. It assesses whether students are “ready” to engage in career problem-solving. In contrast, the CCCS covers the entire career choice process—from exploration and goal setting to decision-making, action-taking, and post-action reflection.

The CCCS and CA (Career Adaptability) are not competing constructs but rather complementary constructs. Career adaptability (Savickas, 2005, 2013), as a concept, acts as a general resource during life-span development to cope with career transitions. It encompasses concern, control, curiosity, and confidence (the “four Cs”) and asks, “How do I adapt to career changes?” On the other hand, CCCS is specific to certain tasks within a particular context. In the current study, it is specific to Chinese high school students and how they navigate subject and major selections within the Gaokao context. The strong correlation between CCCS and CAAS (r = 0.736) shows that the two concepts are related but still have enough variance to be considered distinct constructs.

### 4.3. Comparison with Existing Related Scales in the Chinese Context

Beyond conceptual distinctions from Western constructs, comparing the CCCS with existing career-related scales developed or validated for Chinese high school populations highlights its unique contributions.

Several instruments have been adapted or developed for Chinese students. The Career Decision Self-Efficacy Scale-Short Form (CDSES-SF; Betz & Luzzo, 1996) demonstrated good reliability (α = 0.93) in a Chinese sample (Hampton, 2006). However, it does not take into account the structural constraints of China’s educational system. The Major Decision-Making Self-Efficacy Scale (MDMSES; Li et al., 2025) was developed specifically for Chinese high school students under the New Gaokao reform, comprising five factors (Information Acquisition, Decision Persistence, Self-Determination, Self-Evaluation, and Social Support). However, it remains focused on confidence rather than competence and excludes behavioral action-taking and post-decision reflection. The Career Adapt-Abilities Scale-China Form (CAAS-China Form; Hou et al., 2012) demonstrated a factor structure highly consistent with the international version, which indicates that career adaptability may be relatively universal across different cultures. However, it is worth noting that the Career Curiosity subscale of the CAAS exhibited a Cronbach’s alpha of 0.642 in our sample, consistent with documented reliability variability of the CAAS across samples (Xu et al., 2025). In addition, its four-factor structure addresses broad adaptive capacities rather than the specific, time-constrained choice tasks Chinese high school students face.

Compared with these instruments, the CCCS offers several distinctive contributions: (1) it assesses perception competence rather than confidence (unlike CDSES-SF and MDMSES); (2) it is task-specific to subject and major selection rather than measuring general adaptability (unlike CAAS-China Form); (3) it uniquely incorporates learning efficacy as a culturally relevant dimension reflecting the strong relationship between academic pressure and career choice in Chinese secondary education; (4) it includes self-reflection as a meta-cognitive component largely absent from existing scales. These comparisons reveal that while Western-derived scales have provided valuable starting points, the CCCS represents a meaningful advance by integrating culturally and contextually relevant dimensions tailored to the specific demands of the New Gaokao system.

### 4.4. Contextualizing Within China’s New Gaokao Reform

Firstly, the six-dimensional framework highlights some context-specific aspects of career choice competence within the educational reforms in China. The emphasis on environmental exploration is related to the changes that have been made in the choice architecture through the introduction of the New Gaokao system, where students need to navigate flexible subject combinations and major-oriented admissions. Moreover, the recognition of learning efficacy as a unique dimension relates to the reality that the academic performance of a student determines whether he or she is eligible to choose particular subjects and majors. This contextual sensitivity responds to the need for more localized assessment tools in career psychology (W. Fan & Leong, 2016).

The validated version of the CCCS may be used for many different purposes. For instance, it gives educators and counselors an opportunity to use a preliminary assessment instrument that allows them to assess career choice competence across six dimensions and make targeted interventions within a context where career education is not sufficiently integrated (Gu et al., 2020). Secondly, the six-dimensional approach enables the development of career education curricula that address each aspect of the competence and, thus, may help reduce career decision-making problems and anxiety (H. Chen et al., 2021; Du & Jin, 2016). Third, the competencies measured are transferable skills supporting lifelong career self-management in the VUCA career landscape of the 21st century (Bennett & Lemoine, 2014).

### 4.5. Limitations and Future Research Directions

Several limitations should be acknowledged. First, the samples across the three studies were drawn exclusively from Zhejiang Province, an early pilot region for the New Gaokao reform, which may limit the generalizability of the findings to other provinces or educational systems. The scale is thus recommended for use with students in similar educational contexts, and cross-regional validation is needed before broader generalization.

Second, reliance on self-report data may introduce common method variance and social desirability bias. Therefore, future research should combine this approach with objective behavioral measures, such as choice tracking and information search patterns. Additionally, we acknowledge that test–retest reliability, predictive validity, and measurement invariance across subgroups remain to be established in future research.

Third, while the six-factor model demonstrated good fit in Study 3, further validation should examine its relationships with several related constructs (e.g., career decision-making difficulties, career indecision, vocational identity). Additionally, the cross-sectional design of this study does not permit causal inference; therefore, the relationships among the six dimensions of CCC should be tested in future longitudinal research. Until measurement invariance is confirmed, this scale should be used with caution for between-group comparisons in practice.

Three key directions for future research can be identified from this work. First, future studies should examine whether CCCS scores can predict actual career choice outcomes, including decision satisfaction, academic performance in selected subjects, and university admission results. In addition, the temporal stability of CCCS scores over 4-week and longer intervals needs to be assessed. Second, future intervention research should evaluate whether CCCS scores improve after participation in career education programs or counseling. Furthermore, normative data from diverse samples are required before meaningful cut-off scores can be determined for individual-level clinical or guidance applications. Finally, given the growing role of artificial intelligence (AI) in China, its impact on students’ autonomous career choice competence needs further investigation.

## 5. Conclusions

This study is one of the first systematic attempts to conceptualize, construct, and validate a career choice competence scale for Chinese high school students under the backdrop of the New Gaokao reform. Employing a sequential mixed-methods design, we have successfully constructed a six-dimensional model of career choice competence supported by initial psychometric evidence, which consists of self-cognition, learning efficacy, environmental exploration, outcome expectations, self-reflection, and action-taking.

Theoretically, the six-dimensional model is an expansion of SCCT that specifies process-level competencies for students to implement social-cognitive mechanisms in an increasingly uncertain educational context. Also, the six-dimensional model is complementary to career construction theory since it can capture the meta-cognitive and behavioral dimensions of career self-management. From a practical perspective, the CCCS provides educators and counselors with a preliminary assessment instrument to identify students who need additional career education support. However, it is not yet suitable for individual-level screening, diagnostic, or classification purposes; further validation is needed before the CCCS can be used for practical decision-making applications.

Career decisions have become very important in today’s volatile, uncertain, complex, and ambiguous world, especially due to the growing range of educational choices for Chinese high school students. The CCCS will facilitate further studies on the development and mechanisms of career choice competence, which will eventually help improve students’ decision-making skills and facilitate their transition to higher education and the workforce.

## Figures and Tables

**Table 1:** Comparison of related career choice constructs.

Construct	Core Definition	Structural Dimensions
Career decision-making self-efficacy (Taylor & Betz, 1983)	Confidence in one’s ability to complete specific career decision tasks (e.g., self-appraisal, goal selection, problem-solving)	– Self-appraisal – occupational information – goal selection – planning – problem-solving
Career Choice Readiness (Sampson et al., 2000)	Cognitive, affective, and social preparedness to engage in career problem-solving and decision-making	– Cognitive state (self/occupational knowledge) – Affective state – Social state – Decision-making skills – Metacognitive monitoring
Career Adaptability (Savickas, 2013)	Psychosocial resource for coping with current and anticipated career tasks, transitions, and work traumas	– Career concern – Career control – Career curiosity – Career confidence

**Table 2:** Comparison of Model Fit indices in Covariance structure modeling (*n* = 555).

Fit Index	CMIN/DF	GFI	NFI	TLI	IFI	CFI	RMSEA
Fit parameter	<5	>0.9	>0.9	>0.9	>0.9	>0.9	<0.10
M0 (seven-factor)	3.437	0.843	0.845	0.871	0.885	0.884	0.066
M1 (six-factor)	2.677	0.915	0.922	0.941	0.950	0.950	0.055
Competing model index interpretation	Good	Ideal	Ideal	Ideal	Ideal	Ideal	Ideal

**Table 3:** Reliability Coefficients of the Career Choice Competence Scale (*n* = 421).

Reliability Statistic	Cronbach’s α	Cronbach’s α Based on Standardized Items	Number of Items
Total questionnaire	0.932	0.933	24
Self-cognition	0.759	0.760	3
Learning efficacy	0.878	0.880	4
Environmental exploration	0.912	0.911	4
Self-reflection	0.858	0.859	4
Outcome expectations	0.859	0.859	3
Action-taking	0.889	0.890	6

## Data Availability

The data presented in this study are available on request from the corresponding author. The data are not publicly available due to privacy restrictions.

## Appendix A

**Table A1:** Interview Outline for Career Choice Discussions with High School Students.

Subject	Questions
1. The current career choice status	(1) What subjects do you choose (or subjects you plan to choose)? (2) Have you decided on your major after taking the Gaokao? What is it? (3) Have you ever thought about the career you might pursue in the future? If so, what specific field would you like to work in?
2. The career choice process	(1) How do you choose your subjects? (2) How do you approach choosing your major or future career path? (3) What factors can influence your judgment? (4) How can you determine whether this choice is right for you?
3. Assessment of career choice competence	(1) Do you have the ability to make informed career choices? Why? (2) Which people do you think have made successful or suitable choices for their career? How did they do it? (3) If high school students have nice career choice competence, what specific aspects does it include?

**Table A2:** Example of the conceptualization process for open coding.

No.	Data Text (Original Data)	Open Coding	Axial Coding (Category)	Selective Coding (Core Category)
1	a3: Subjects like geography and technology are relatively easier to learn; with the same amount of time invested, you’re likely to achieve significantly higher scores.	Learning input-output ratio	Learning efficacy	Self-exploration
4	a4: I have a strong interest in history, while politics interests me because I enjoy studying the ideologies that emerged across different historical periods and regions worldwide.	Subject preference	Personal interest	
8	a9: I chose chemistry mainly because I want to pursue a science-related major in the future, and there are slightly more excellent programs available in this field.	Breadth of major choices	Environmental requirements	Environmental exploration
10	a12: I chose the medical field because, in addition to becoming a physician, I can also engage in scientific research or work at pharmaceutical companies.	Major development path	Major understanding	
19	a22: I am absolutely determined about the subjects I choose; my choice won’t change just because I perform well or poorly on a single exam.	Goal commitment after choosing	Goal commitment	Outcome expectations
24	a32: While making independent decisions is crucial, I believe it’s essential to consider the opinions of parents and others as well—relying solely on personal assumptions simply won’t suffice.	Integrating own and others’ opinions	Comprehensive weighing and analysis	Decision-making participation
28	a37: I believe that compared to academic performance, the ability to make choices is actually more important, as it is sustainable and can prove valuable in various other aspects.	Reflecting on choice ability	Reflection on choice	Self-reflection
29	a49: I generally have a good ability to manage my time and can clearly plan what I need to do now and what comes next.	Good time management and planning	Discipline and proactive engagement	Action-taking

**Table A3:** Distribution of coding nodes for psychological connotation of career choice competence (*n* = 40).

No.	Open Coding Node	Encoding Definition	Reference Points	Axial Coding (Category)	Selective Coding (Core Category)
1	Considering subjects of strength	Students’ comprehensive beliefs about their ability to achieve academic goals	32	Learning efficacy	Self-exploration
2	Thinking style and learning feel	Students’ confidence level in their ability to achieve learning goals for a particular subject	7		
3	Learning input-output ratio	Students’ estimates of the efficiency of their academic input and output	11		
4	Subject preference	A clear understanding and description of students’ interests and preferences in their subjects	36	Personal interest	
5	Major interest	A clear understanding and description of students’ preferences and interests in their major	13		
6	Vocational inclination	A clear understanding and description of students’ preferences for their career interests	10		
7	Ability and strengths	Students’ objective judgment of their ability strengths and weaknesses	11	Ability and strengths	
8	Subject selection requirements	Students’ understanding of the new college entrance examination subject selection policy and admission rules	9	Environmental requirements	Environmental exploration
9	Breadth of major choices	The behavior of students actively collecting information about the range of major choices	7		
10	Good employment prospects	Students’ proactive engagement in exploring employment prospects	11	Major understanding	
11	Major curriculum content	Students’ proactive engagement in seeking information about relevant universities and majors	5		
12	Major development path	Students’ proactive engagement in exploring their professional career paths	6		
13	Job content	Students’ proactive engagement in learning about career-related topics	3	Occupation understanding	
14	Work values	Students actively explore the value of work	4		
15	Job income and stability	Students’ proactive behavior in seeking information about job income and stability	6		
16	Societal development needs	Students’ proactive behaviors aimed at understanding the needs of social development	5	Social development prospects	
17	Social employment prospects	Students’ proactive engagement in exploring social employment prospects	9		
18	Educational requirements and development space	Students’ proactive engagement in understanding the educational requirements and career development opportunities	4		
19	Having clear subject selection goals	Students have clear subject selection goals	35	Goal clarity	Outcome expectations
20	Having clear major goals	Students have clear major selection goals	21		
21	Having clear career goals	Students have clear vocation selection goals	9		
22	Goal commitment after choosing	The persistence level and inflexibility of students’ goal intentions	15	Goal commitment	
23	Goals are suitable for me	The rationale and logic upon which students base their goal-setting decisions	15	Goal suitability	
24	Obtaining major information from official channels	Students’ behavior of obtaining professional decision-making information through official channels	6	Information acquisition	Decision-making participation
25	Collecting major information online	Students’ online decision-making information-seeking behavior	9		
26	Getting major information from family and friends	The behavior of students obtaining decision-making information from family members or friends	12		
27	Consulting parents’ opinions	Students actively seeking their parents’ opinions	17	Proactive communication	
28	Asking for opinions from senior students/classmates	Students actively seeking advice from their senior peers	5		
29	Asking for opinions from teachers and others	Students’ proactive behavior in communicating with teachers and others.	10		
30	Considering ranking methods	Students actively consider decision-making ranking methods	14	Considering ranking methods	
31	Integrating personal and environmental conditions	The student’s ability to comprehensively weigh various types of information	27	Comprehensive weighing and analysis	
32	Integrating own and others’ opinions	The student’s ability to integrate their own opinions with those of others	21		
33	Making autonomous decisions	Students’ tendency to make subject and career choices independently	18	Making decisions	
34	Deciding after consulting others’ opinions	Students’ tendency to make decisions after consulting others’ opinions	26		
35	Reflecting on my own positioning	Students reflect on their own mental state	9	Self-reflection on choice	Self-reflection
36	Reflecting on the psychological state of choice	Students reflect on their selection process	5		
37	Reflecting on choice ability	Students reflect on their ability to make choices	15		
38	Independent thinking ability	Students reflect on their capacity for independent thinking	6		
39	Increased awareness of career planning	Students have developed a greater awareness of career planning	14	Reflection on choice awareness	
40	Subjective initiative awareness	Students possess an active awareness of making career choices.	9		
41	Reflecting on the steps of choice	Students reflect on the steps involved in making career choices	9	Reflection on choice process	
42	Reflecting on the suitability of the choice	Students reflect on the suitability of their choices	13		
43	Reflecting on the goal-orientation of the choice	Students reflect on their personal goals and direction	8		
44	Reflecting on factors influencing the choice	Students reflect on the factors influencing their choices	7		
45	Self-adjustment after making a choice	Students’ self-regulatory behaviors after making a choice	5	Adjustment and adaptation	Action-taking
46	Considering alternative options	Students have a range of alternative options to consider	5		
47	Considering changing career direction	Students are considering changing their career path	4		
48	Taking responsibility for choices	Students’ assumption of responsibility for their choices	4	Discipline and proactive engagement	
49	Good time management and planning	Students’ behavior in time management and planning	7		
50	Making efforts with focus	The student’s behavior of maintaining focus after making a choice	5		
51	Self-discipline	Students’ self-disciplined behavior after making a choice	4		
52	Being action-oriented	Students’ behavior to overcome difficulties and persist in their efforts during the implementation process	6		

**Table A4:** Item Analysis Results.

Item	Extreme-Group Comparison	General Relevance (Item Scores Are Correlated with the Total Score)	Homogeneity Test (α Reliability After Item)	Items Not Meeting the Standard Indicators	Selection
Judgment Criteria	≥3.000	≥0.400	≤0.938		
X1	4.419	0.280 **	0.938	r < 0.400	delete
X2	8.101	0.480 **	0.937		remain
X3	4.175	0.419 **	0.938		remain
X4	7.043	0.526 **	0.937		remain
X5	6.334	0.338 **	0.938	r < 0.400	delete
X6	7.225	0.464 **	0.937		remain
X7	7.217	0.501 **	0.937		remain
X8	7.598	0.502 **	0.937		remain
X9	5.682	0.449 **	0.937		remain
X10	8.658	0.524 **	0.937		remain
X11	2.895	0.260 **	0.938	t < 3.000; r < 0.400	delete
X12	11.683	0.635 **	0.936		remain
X13	11.036	0.593 **	0.936		remain
X14	11.667	0.647 **	0.936		remain
X15	12.179	0.679 **	0.936		remain
X16	14.480	0.719 **	0.935		remain
X17	7.755	0.559 **	0.937		remain
X18	11.724	0.662 **	0.936		remain
X19	4.359	0.346 **	0.938	r < 0.400	delete
X20	8.550	0.563 **	0.937		remain
X21	7.251	0.413 **	0.938		remain
X22	10.046	0.622 **	0.936		remain
X23	7.148	0.377 **	0.938	r < 0.400	delete
X24	9.731	0.602 **	0.936		remain
X25	11.349	0.647 **	0.936		remain
X26	8.642	0.574 **	0.937		remain
X27	9.608	0.642 **	0.936		remain
X28	5.515	0.430 **	0.937		remain
X29	3.803	0.342 **	0.938	r < 0.400	delete
X30	7.398	0.493 **	0.937		remain
X31	8.061	0.526 **	0.937		remain
X32	6.223	0.514 **	0.937		remain
X33	4.263	0.239 **	0.939	r < 0.400; α > 0.938	delete
X34	3.854	0.313 **	0.938	r < 0.400	delete
X35	9.380	0.561 **	0.937		remain
X36	4.797	0.279 **	0.939	r < 0.400; α > 0.938	delete
X37	6.131	0.461 **	0.937		remain
X38	11.629	0.616 **	0.936		remain
X39	10.585	0.631 **	0.936		remain
X40	9.739	0.556 **	0.937		remain
X41	7.120	0.372 **	0.938	r < 0.400	delete
X42	7.710	0.494 **	0.937		remain
X43	4.699	0.313 **	0.938	r < 0.400	delete
X44	9.420	0.552 **	0.937		remain
X45	9.527	0.582 **	0.936		remain
X46	6.873	0.574 **	0.937		remain
X47	8.305	0.620 **	0.936		remain
X48	8.779	0.598 **	0.936		remain
X49	2.321	0.138 **	0.940	t < 3.000; r < 0.400; α > 0.938	delete
X50	8.137	0.519 **	0.937		remain

Note: ** *p* < 0.01.

**Table A5:** Exploratory Factor Analysis component loadings and commonality (*n* = 265).

	Item	Factor 1	Factor 2	Factor 3	Factor 4	Factor 5	Factor 6	Factor 7	Commonality
X1	I know my personality strengths.	**0.746**	0.182	0.097	0.121	0.044	0.183	0.041	0.480
X3	I know what abilities I am good at.	**0.581**	0.392	0.214	0.081	−0.028	0.111	0.107	0.524
X2	I know what kind of life I want to live.	**0.488**	0.134	0.246	0.207	0.121	−0.051	0.082	0.464
X7	I know which subjects are easier for me to learn.	0.102	**0.865**	0.094	0.072	0.106	0.094	0.119	0.502
X6	I know which subjects I have better grades in.	0.244	**0.801**	0.056	0.193	0.038	0.077	0.052	0.501
X8	I know whether I am better at logical or verbal reasoning.	0.071	**0.749**	0.098	0.004	0.137	0.095	0.110	0.449
X5	I am clear about which subjects I am more interested in learning.	0.179	**0.736**	0.146	0.156	0.167	0.110	−0.017	0.526
X12	I will look for information about university majors that I might be interested in.	0.118	0.103	**0.830**	0.135	0.104	0.200	0.108	0.679
X13	I will look for information about universities I might want to apply to.	0.172	0.115	**0.825**	0.192	0.109	0.209	0.109	0.719
X11	I will browse information on university disciplines and major classifications.	0.075	0.044	**0.824**	0.134	0.233	0.028	0.160	0.647
X9	I will search for the subject requirements of different universities and majors.	0.058	0.086	**0.812**	0.100	0.093	0.138	0.182	0.635
X18	I often think about what direction is suitable for me.	0.100	0.102	0.219	**0.805**	0.251	0.138	0.092	0.620
X17	I often think about what kind of person I am, and what my strengths and weaknesses are.	0.197	0.035	0.130	**0.767**	0.175	0.118	0.159	0.574
X19	I often think about what I need to do next.	0.137	0.203	0.182	**0.751**	0.024	0.082	0.229	0.598
X15	I often think about what type of career might suit me.	0.055	0.072	0.377	**0.558**	0.197	0.186	−0.023	0.559
X29	If I find that a selected subject is not suitable for me, I can come up with a solution.	0.030	0.062	0.240	0.163	**0.702**	0.053	0.153	0.526
X28	Making a choice makes me feel that my learning purpose is clearer and more meaningful.	−0.055	0.160	0.263	0.157	**0.664**	−0.067	0.140	0.493
X33	I face the challenges of subject and major selection with a positive attitude.	0.167	0.182	0.000	0.054	**0.531**	0.350	0.210	0.519
X22	I feel there is a high probability that I can be admitted to a major I like.	0.236	0.071	0.227	0.148	0.180	**0.710**	0.121	0.622
X23	I feel there is a high probability that I can engage in a career I like in the future.	0.284	0.070	0.107	0.198	0.273	**0.665**	0.133	0.602
X21	I feel there is a high probability that I can learn the subjects I have chosen well.	−0.111	0.337	0.223	0.156	0.067	**0.580**	0.228	0.563
X32	To achieve my goals, I will adjust my learning methods to improve my grades.	−0.016	0.157	0.233	0.132	0.074	0.127	**0.781**	0.556
X31	To achieve my goals, I will plan and manage my time well.	0.081	0.016	0.226	0.171	0.266	0.183	**0.777**	0.631
X30	To achieve my goals, I will create a study plan that suits me.	0.122	0.053	0.261	0.095	0.257	0.142	**0.774**	0.616

**Table A6:** Corresponding indicators for each dimension of the Six-Factor Model (*n* = 555).

			Estimate	S.E.	C.R.	*p*	Standardization Coefficient	AVE	CR
X3	<---	F1	1.000				0.823	0.5215	0.7627
X2	<---	F1	0.655	0.051	12.891	***	0.594		
X1	<---	F1	0.756	0.049	15.460	***	0.731		
X8	<---	F2	1.000				0.614	0.6685	0.8876
X7	<---	F2	1.310	0.080	16.316	***	0.904		
X6	<---	F2	1.199	0.074	16.305	***	0.903		
X5	<---	F2	1.141	0.075	15.296	***	0.815		
X13	<---	F3	1.000				0.860	0.6810	0.8937
X12	<---	F3	1.091	0.038	28.453	***	0.945		
X11	<---	F3	0.889	0.037	23.719	***	0.809		
X9	<---	F3	0.677	0.039	17.496	***	0.661		
X19	<---	F4	1.000				0.705	0.5777	0.8428
X18	<---	F4	1.253	0.067	18.651	***	0.908		
X17	<---	F4	1.047	0.062	17.024	***	0.784		
X15	<---	F4	0.900	0.067	13.447	***	0.612		
X23	<---	F5	1.000				0.836	0.6423	0.8407
X22	<---	F5	1.083	0.048	22.525	***	0.906		
X21	<---	F5	0.783	0.050	15.806	***	0.638		
X29	<---	F6	1.000				0.634	0.5341	0.8718
X28	<---	F6	1.008	0.079	12.706	***	0.634		
X30	<---	F6	1.163	0.083	14.077	***	0.723		
X31	<---	F6	1.120	0.080	13.917	***	0.722		
X32	<---	F6	1.197	0.077	15.550	***	0.832		
X33	<---	F6	1.249	0.081	15.361	***	0.815		

Note: *** *p* < 0.001.

**Table A7:** Discriminant validity of each factor in the six-factor model (*n* = 555).

Variable	1	2	3	4	5	6
F1	0.722					
F2	0.623	0.818				
F3	0321	0.235	0.825			
F4	0.459	0.445	0.263	0.760		
F5	0.469	0.481	0.311	0.399	0.801	
F6	0.468	0.448	0.356	0.583	0.635	0.731

**Table A8:** Internal consistency coefficients, means, and standard deviations for CCCS and CAAS (*n* = 421).

Questionnaire		Cronbach’s α	M	SD	Number of Items	Total Cronbach’s α
Career Choice Competence Scale (CCCS)	Self-cognition	0.759	3.907	0.815	3	0.932
	Learning efficacy	0.878	3.902	0.889	4	
	Environmental exploration	0.912	2.901	1.073	4	
	Self-reflection	0.858	3.600	0.900	4	
	Outcome expectations	0.859	3.220	0.999	3	
	Action-taking	0.889	2.994	0.853	6	
Career Adapt-Abilities Scale (CAAS)	Career concern	0.803	3.607	0.862	3	0.873
	Career control	0.775	3.833	0.798	3	
	Career curiosity	0.642	3.716	0.765	3	
	Career confidence	0.808	3.660	0.798	3	

**Table A9:** Cross-validation: Model Fit indices in Covariance structure modeling (*n* = 421).

Fit Index	CMIN/DF	GFI	NFI	TLI	IFI	CFI	RMSEA
Fit parameter	<5	>0.9	>0.9	>0.9	>0.9	>0.9	<0.10
Fitting Indicator	2.707	0.892	0.903	0.926	0.937	0.937	0.064
Index interpretation	Ideal	Marginal	Ideal	Ideal	Ideal	Ideal	Ideal

**Table A10:** Correlation between CCCS and CAAS (*n* = 421).

Questionnaire	Career Concern	Career Control	Career Curiosity	Career Confidence	Career Adaptability Total Score
Self-cognition	0.429 **	0.437 **	0.413 **	0.423 **	0.541 **
Learning efficacy	0.420 **	0.441 **	0.425 **	0.441 **	0.549 **
Environmental exploration	0.434 **	0.242 **	0.257 **	0.229 **	0.374 **
Self-reflection	0.594 **	0.442 **	0.540 **	0.469 **	0.652 **
Outcome expectations	0.438 **	0.413 **	0.371 **	0.453 **	0.534 **
Action-taking	0.516 **	0.431 **	0.448 **	0.554 **	0.621 **
Career choice competence total score	0.643 **	0.534 **	0.550 **	0.580 **	0.736 **

Note: ** *p* < 0.01.

**Table A11:** The final version of Competence Choice Competence (CCCS).

	Item	Factor	M	SD
1	I know my personality strengths.	Self-cognition	3.900	0.782
2	I know what abilities I am good at.			
3	I know what kind of life I want to live.			
4	I am clear about which subjects I am more interested in learning.	Learning efficacy	4.040	0.824
5	I know which subjects I have better grades in.			
6	I know which subjects are easier for me to learn.			
7	I know whether I am better at logical or verbal reasoning.			
8	I have researched what the subject selection requirements are for different universities and majors.	Environmental exploration	2.837	1.022
9	I have browsed information on university disciplines and major classifications.			
10	I have looked up information about university majors that might interest me.			
11	I have looked for information regarding the universities I might want to apply to.			
12	I often think about what type of career might be suitable for me.	Self-reflection	3.612	0.651
13	I often think about what kind of person I am, and what my strengths and weaknesses are.			
14	I often think about what direction is suitable for me.			
15	I often think about what I need to do next.			
16	I feel there is a high probability that I can learn the subjects I have chosen well.	Outcome expectations	3.500	0.888
17	I feel there is a high probability that I can be admitted to a major I like.			
18	I feel there is a high probability that I can engage in a career I like in the future.			
19	Making a choice makes me feel that my learning purpose is clearer and more meaningful.	Action-taking	3.224	0.804
20	If the choices I make are not right for me, I know how to handle it.			
21	To achieve my goals, I will create a study plan that suits me.			
22	To achieve my goals, I will plan and manage my time well.			
23	To achieve my goals, I will adjust my learning methods to improve my grades.			
24	I face the challenges of subject and major selection with a positive attitude.			
